# Supplementary material for: Resectability and Ablatability Criteria for the Treatment of Liver Only Colorectal Metastases: Multidisciplinary Consensus Document from the COLLISION Trial Group
Source: Cancers (Basel). 2020 Jul 3;12(7):1779. doi: 10.3390/cancers12071779 (PMC7407587; doi:10.3390/cancers12071779)
Supplement: Supplementary file 1 [file cancers-12-01779-s001.zip › File S3 - statements plus outcomes.pdf]

Online appendix C: Statements plus outcomes

1. AGE: Curative-intent local therapy for CRLM is appropriate for all age groups at the prerequisite that the general health status is adequate (ECOG ≤2, ASA ≤3 and CCI ≤8); Treatment cannot be witheld based on patients' age alone	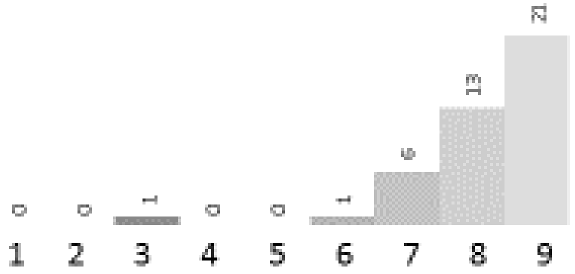
2. EASTERN COOPERATIVE ONCOLOGY GROUP (ECOG) SCORE: Curative-intent local therapy for CRLM is appropriate for ECOG ≤2 patients, for ECOG ≥3 patients the risks of surgery, thermal ablation and IRE do not outweigh the benefits; in select patients with limited disease (≤3 CRLM) SBRT can be considered for ECOG 3 patients with a life expectancy >1 year	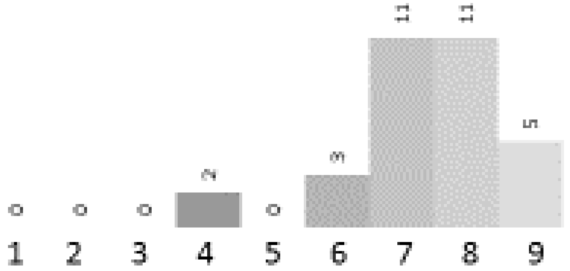
3. AMERICAN SOCIETY of ANESTHESIOLOGISTS (ASA) SCORE: Curative-intent local therapy for CRLM is appropriate for ASA ≤3 patients, for ASA 4 patients the risks of surgery, thermal ablation and IRE do not outweigh the benefits; in select patients with limited disease (≤3 CRLM) SBRT can be considered for ASA 4 patients	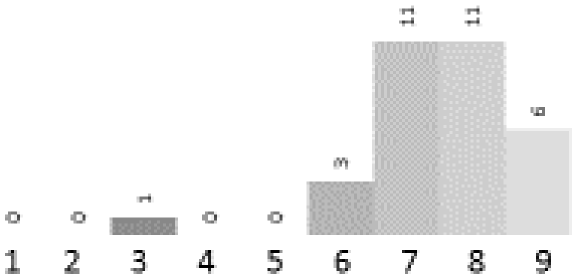
4. UNDERLYING LIVER DISEASE (ICD-10): Curative-intent local therapy for CRLM is appropriate for no or mild underlying liver disease; for severe underlying liver disease the risks do not outweigh the benefits.	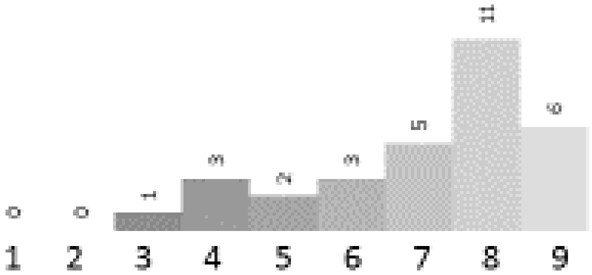

5. CHARLSON COMORBIDITY INDEX (CCI): Curative-intent local therapy for CRLM is appropriate for patients with CCI ≤4 and for patients with CCI 5-8 if the procedure is considered non-complex (minor hepatectomy +/- ablations); for patients with a CCI ≥9 the risks of surgery, thermal ablation and IRE do not outweigh the benefits; in select patients with limited disease (≤3 CRLM) SBRT can be considered if CCI is 9-10.	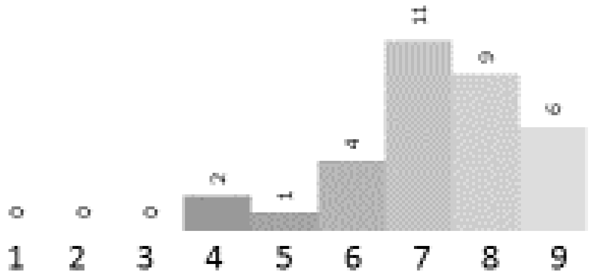
6. STAGE IVa DISEASE: Upfront curative intent local treatment without peri-procedural systemic therapy is the appropriate therapy if the procedure is considered non-complex (minor hepatectomy and/or ablations).	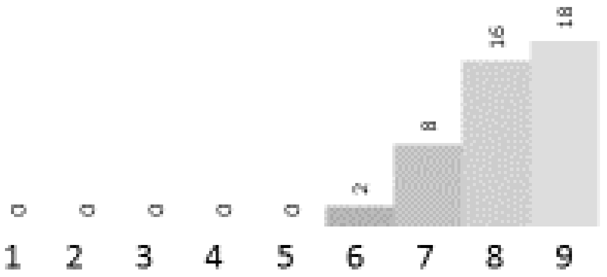
7. STAGE IVb DISEASE: Upfront curative intent local treatment without peri-procedural systemic therapy is the appropriate therapy if the surgical procedure, with or without ablative treatment, is considered complex (major hepatectomy +/- ablations), with the following two exemptions where 4-6 cycles of induction systemic therapy are indicated: (a) if downsizing systemic therapy is likely to reduce the surgical risk or (b) in case of early metachronous disease developed within 6 months following primary tumor diagnosis (test of tumor biology).	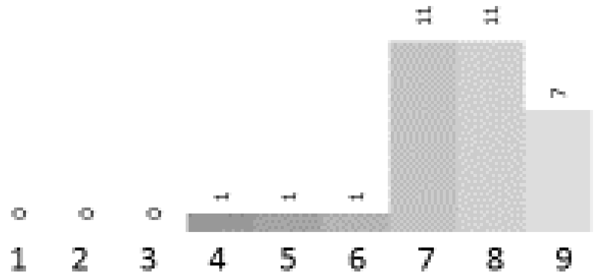
8. STAGE IVc DISEASE: In patients, unsuitable for curative intent surgery and/or ablation due to number, size and location of CRLM, with potentially downstagable disease, induction systemic therapy is appropriate until: a) curative intent local treatment has become possible or (b) when	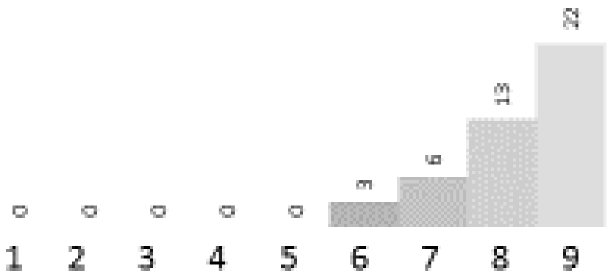

further downsizing will not (further) decrease procedural risk.	
9. STAGE IVd DISEASE: For liver-only colorectal metastases, the term permanently unsuitable for curative intent local treatment should be reserved for (a) patients who remain ineligible for radical intent local therapy following induction systemic therapy, (b) patients with upfront contra-indications for radical intent local therapy and contra-indications to receive systemic therapy and (c) patients with a poor general health status who do not qualify for any local therapy (SBRT, local ablation and surgery).	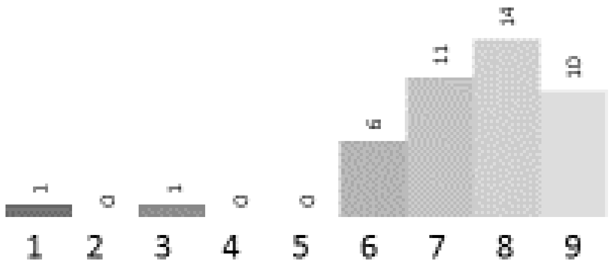
10. PROGNOSTIC BIOMARKERS: At the prerequisite that the primary tumor plus any locoregional lymph nodes are (or will be) radically resected (or treated with radical intent otherwise), curative intent local treatment can currently not be (dis)qualified or classified to specific local treatment groups based on the following parameters: primary tumor location, synchronous versus metachronous disease, previous (neo)adjuvant therapies for locoregional disease, the best objectified respons to systemic treatment, (y)p/cT-stage and (y)p/cN-stage, RAS or BRAF wildtypes or mutations, microsatellite (in)stability, consensus molecular subtypes, clinical risk score (CRS by Fong et al.) and the modified CRS, CEA or other tumor marker quantities, the presence and quantity of circulating tumor cells and DNA.	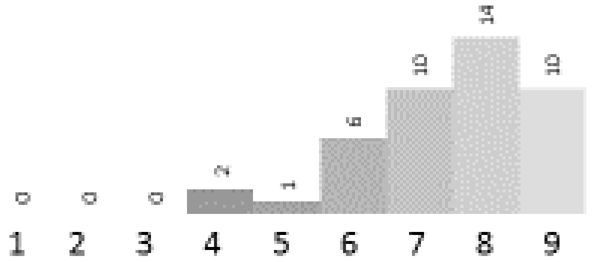

11. FUTURE LIVER REMNANT (FLR) VOLUME AND/OR FUNCTION: If the FLR volume and/or function is sufficient, curative-intent local therapy for CRLM is appropriate, regardless of the total number of CRLM; patients cannot be disqualified based on the total number of CRLM alone.	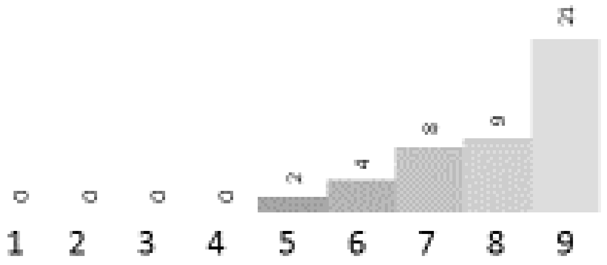
12. Partial hepatectomy is the appropriate local treatment method for patients with resectable CRLM >3cm, at the prerequisite that ECOG is ≤2, ASA is ≤3 and CCI is ≤8.	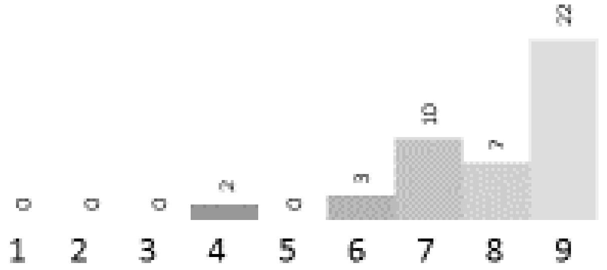
13. Partial hepatectomy is the appropriate local treatment method for patients with resectable exophytic or perihilar CRLM ≤3cm, at the prerequisite that ECOG is ≤2, ASA is ≤3 and CCI is ≤8.	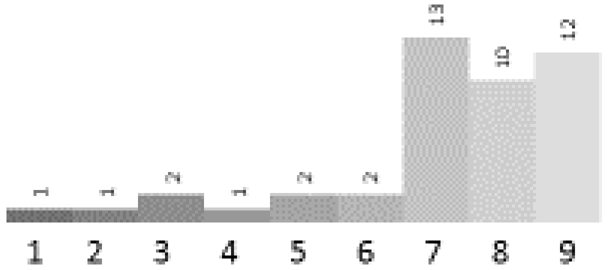
14. Partial hepatectomy is the appropriate local treatment method for patients with resectable superficial or shallow CRLM ≤3cm, at the prerequisite that the general health status is <u>good</u> (ECOG ≤1, ASA ≤3 and CCI ≤4).	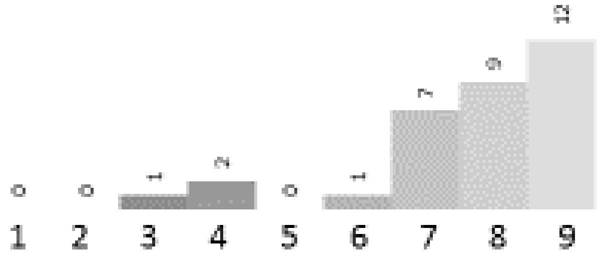

15. Thermal ablation can be considered for patients with resectable superficial or shallow CRLM ≤3cm, if the general health status is *<u>poor</u> (ECOG 2 and ASA 3 or CCI 5-8).*	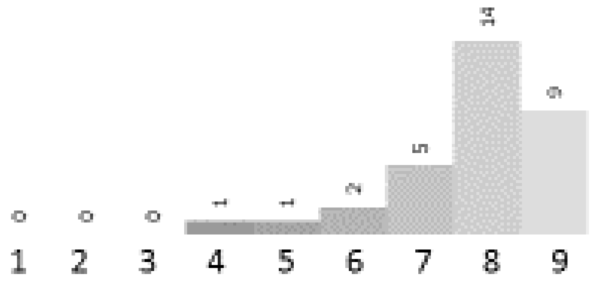
16. Thermal ablation is the appropriate local treatment method for patients with resectable and thermally ablatable CRLM ≤3cm, if the location of the CRLM is deep-seated (e.g. resection would require major hepatectomy), at the prerequisite that ECOG is ≤2, ASA is ≤3 and CCI is ≤8).	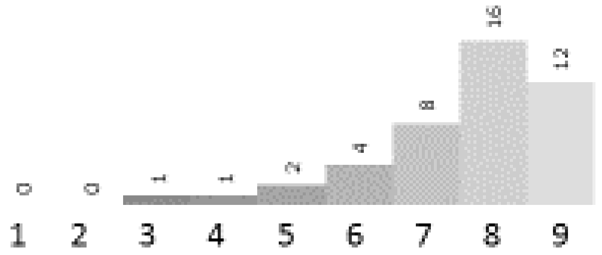
17. Thermal ablation is the appropriate local treatment method for patients with unresectable and thermally ablatable CRLM ≤3cm, and can be considered for CRLM 3-5cm when (further) downsizing systemic therapy is unfeasible, at the prerequisite that CCI ≤8, ASA is ≤3 and ECOG is ≤2.	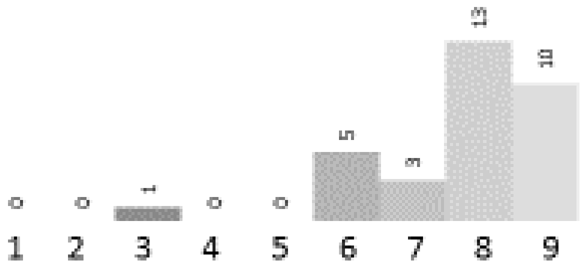
18. Irreversible electroporation (IRE) can be considered for patients with unresectable and not thermally ablatable perihilar or perivascular CRLM ≤3cm, and 3-5cm if further downsizing systemic therapy is unfeasible, at the prerequisite that CCI is ≤8, ASA is ≤3 and ECOG is ≤2.	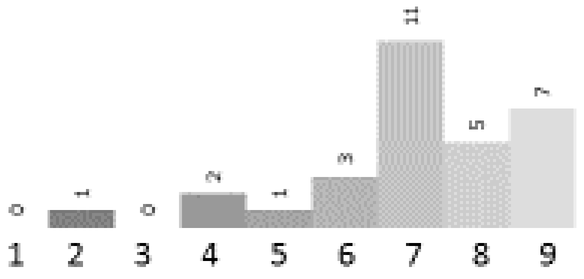

19. Stereotactic ablative radiotherapy (SBRT) can be considered for patients with limited disease burden (≤3 CRLM) if an ablative dose can be delivered without jeopardizing liver function and other organs or structures at risk, at the prequisite that ECOG is ≤3, ASA is ≤4 and CCI is ≤10.	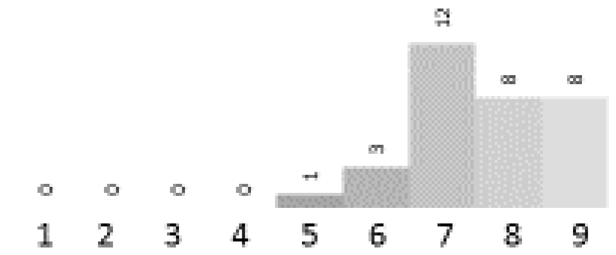
20. Hemihepatectomy is the appropriate local treatment method for multiple CRLM (≥3) within a single lobe when at least one of these CRLM is deep-seated, even when potentially ablatable, at the prerequisite that CCI is ≤8, ASA is ≤3 and ECOG is ≤2.	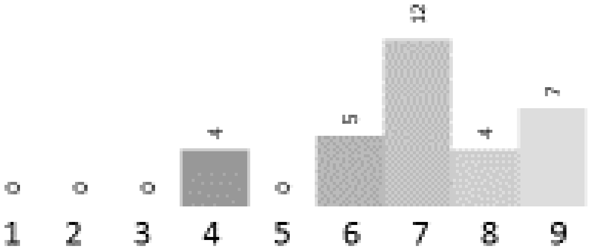
21. When considering fit patients with multiple scattered and bilobar CRLM ≤3cm (≥6 CRLM in total and ≥3 deep-seated CRLM in both lobes separately) what treatment is appropriate: chip-and-burn wedge resection(s) of all exophytic, superficial and shallow CRLM and thermal ablation of all deep-seated CRLM; or a 2-stage-hepatectomy: stage 1: wedge resection(s) of all superficial CRLM and thermal ablations of all deep-seated CRLM in 1 lobe and (following contralateral liver augmentation) stage 2: contralateral hemihepatectomy.<br><br>*Score 1-3: chip-and-burn*<br>*Score 4-6: equipoise*<br>*Score 7-9: 2-stage-hepatectomy*	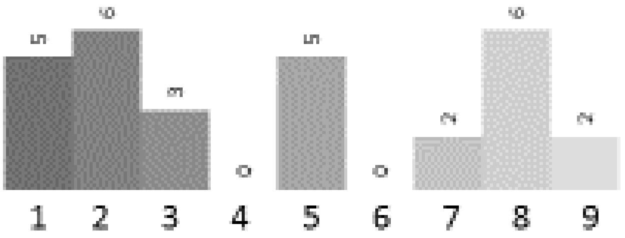

<table>
<tr>
<td>22. Anatomical contra-indications for partial hepatectomy are: (a) inability to obtain R0 margins, (b) inability to leave a sufficient FLR volume and/or function, (c) inability to preserve the dual blood supply and the venous and biliary drainage from the FLR and (d) inaccessibility of the abdominal cavity due to excessive abdominal adhesions.</td>
<td>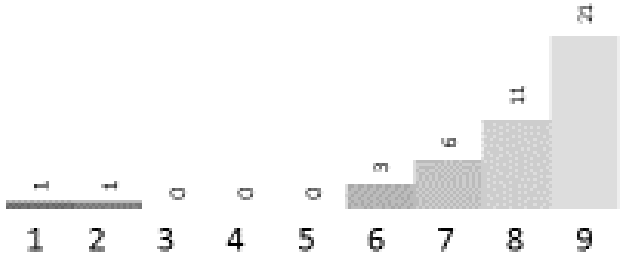
</td>
</tr>
<tr>
<td>23. Anatomical contra-indications for thermal ablation are: (a) peri-tumoral vicinity (<10mm) of the common, left or right hepatic bile duct or (b) peri-hepatic critical structures that cannot be distanced using surgical or interventional dissection methods, (c) the abutment or encasement of the single remaining major portal or systemic vein following surgery and (d) an invasion of the free wall of the inferior caval vein. The maximum size is 3cm, although thermal ablation can be considered for 3-5cm unresectable CRLM after failure to (further) downsize them with systemic therapy.</td>
<td>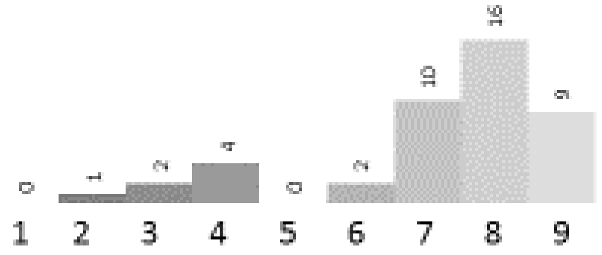
</td>
</tr>
<tr>
<td>24. Contra-indications for irreversible electroporation are: CRLM >5cm, ventricular arrhythmias, cardiac stimulation devices and congestive heart failure.</td>
<td>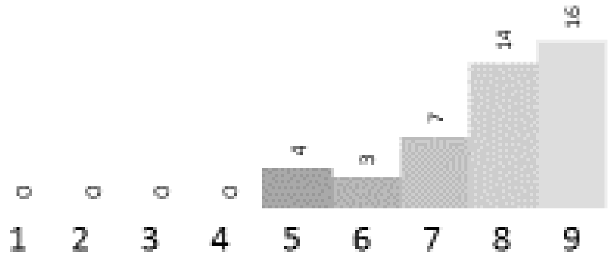
</td>
</tr>
<tr>
<td>25. Contra-indications for stereotactic body radiotherapy (SBRT) are: >3 CRLM and inability to deliver an ablative dose without jeopardizing liver function and adjacent organs or structures at risk.</td>
<td>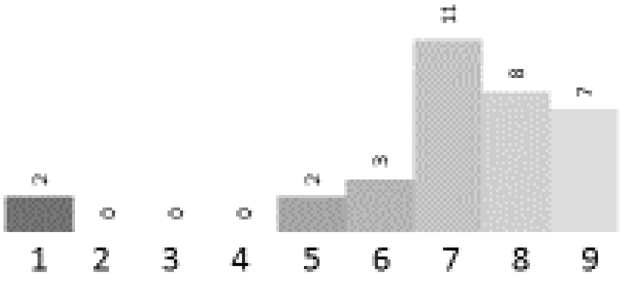
</td>
</tr>
</table>
