# Supplementary material for: Resectability and Ablatability Criteria for the Treatment of Liver Only Colorectal Metastases: Multidisciplinary Consensus Document from the COLLISION Trial Group
Source: Cancers (Basel). 2020 Jul 3;12(7):1779. doi: 10.3390/cancers12071779 (PMC7407587; doi:10.3390/cancers12071779)
Supplement: Supplementary file 1 [file cancers-12-01779-s001.zip › File S1 - statements.pdf]

Online appendix A: Statements

PATIENT CHARACTERISTICS

1. AGE: Curative-intent local therapy for CRLM is appropriate for all age groups at the prerequisite that the general health condition is adequate (ECOG ≤2, ASA ≤3 and CCI ≤8); Treatment cannot be witheld based on patients' age alone [1-4].

   Based on several comparative retrospective studies, that have used matching or multivariate analysis, overall survival was worse in elderly patients compared to younger patients. However, a substantial proportion of elderly patients did achieve long-term disease-free and overall survival and curative intent local therapies were superior to chemotherapy alone in this subset of patients. Hence, elderly patients should not be excluded from receiving curative intent local treatments.

   EVIDENCE LEVEL LOW-MODERATE: SEVERAL RETROSPECTIVE COMPARATIVE CASE SERIES AND LARGER REGISTRY RESULTS

2. EASTERN COOPERATIVE ONCOLOGY GROUP (ECOG) SCORE: Curative-intent local therapy for CRLM is appropriate for ECOG ≤2 patients, for ECOG ≥3 patients the risks of surgery, thermal ablation and IRE do not outweigh the benefits[5-8]; in select patients with limited disease (≤3 CRLM) SBRT can be considered for ECOG 3 patients with a life expectancy >1 year[9-11].

   Based on several studies, 3-month postoperative mortality of surgery +/- ablations was very high in patients with an ECOG score of 3 or 4 (29.6 and 57.6% respectively) compared to patients with an ECOG score of 0-1 or 2 (2.6 and 3.7% respectively). Consequently, for ECOG ≥3 the risks of surgery do not outweigh the benefits.

   EVIDENCE LEVEL LOW-MODERATE: SEVERAL RETROSPECTIVE COMPARATIVE CASE SERIES AND LARGER REGISTRY RESULTS

   Based on a limited number of studies, liver SBRT, which does not require anesthesia or conscious sedation, seems to be safe and locally effective in select patients with a poor performance status.

   EVIDENCE LEVEL LOW: SMALL NON COMPARATIVE CASE SERIES & EXPERT OPINIONS

3. AMERICAN SOCIETY of ANESTHESIOLOGISTS (ASA) SCORE: Curative-intent local therapy for CRLM is appropriate for ASA ≤3 patients, for ASA 4 patients the risks of surgery, thermal ablation and IRE do not outweigh the benefits; in select patients with limited disease (≤3 CRLM) SBRT can be considered for ASA 4 patients[8-13].

   Based on several studies ASA ≥4 was associated with a higher mortality (15.3 – 43.2%) and severe morbidity (37.5%).

   EVIDENCE LEVEL LOW-MODERATE: SEVERAL RETROSPECTIVE COMPARATIVE CASE SERIES AND LARGER REGISTRY RESULTS

4. UNDERLYING LIVER DISEASE (ICD-10): Curative-intent local therapy for CRLM is appropriate for no or mild underlying liver disease; for severe underlying liver disease the risks do not outweigh the benefits [14,15].

   The overall surgical risk of mortality for patients with severe underlying liver disease is ≥40%. The limited data available specifically for liver surgery suggest that the risk is even higher. Hence, curative intent local therapy should not be performed for patients with severe underlying liver disease. EVIDENCE LEVEL LOW: RETROSPECTIVE COMPARATIVE CASE SERIES AND REGISTRY RESULTS

5. CHARLSON COMORBIDITY INDEX (CCI): Curative-intent local therapy for CRLM is appropriate for patients with CCI ≤4 and for patients with CCI 5-8 if the procedure is considered non-complex (minor hepatectomy +/- ablations); for patients with a CCI ≥9 the risks of surgery, thermal ablation and IRE do not outweigh the benefits; in select patients with limited disease (≤3 CRLM) SBRT can be considered if CCI is 9-10 [8-10,16,17].

   Based on our literature review the 3-month mortality after surgery is 3.4-7.2% for CCI 0-2, 18.5% for CCI 3-4 and 33.8% for CCI 5-8.

   EVIDENCE LEVEL LOW-MODERATE: SEVERAL RETROSPECTIVE COMPARATIVE CASE SERIES AND LARGER REGISTRY RESULTS

   Based on a limited number of studies, liver SBRT, which does not require anesthesia or conscious sedation, seems to be safe and locally effective in select patients with a poor performance

   EVIDENCE LEVEL LOW: SMALL NON COMPARATIVE CASE SERIES & EXPERT OPINIONS

DISEASE CHARACTERISTICS

6. STAGE IVa DISEASE: Upfront curative intent local treatment without peri-procedural systemic therapy is the appropriate therapy if the procedure is considered *non-complex* (minor hepatectomy and/or ablations) [18,19].

   The results from the large phase III RCT, where the addition of perioperative chemotherapy (FOLFOX4) over surgery alone for patient with resectable CRLM was assessed, found a marginal difference in disease-free survival and concluded that the primary endpoint was met. However, when analyzing the long term results no overall survival benefit was found. The majority of guidelines state that, because the initial primary endpoint was met, periprocedural systemic therapy should be offered. Others, including the Dutch IKNL guidelines, believe that disease-free survival should merely be regarded as a surrogate endpoint for overall survival and, even though the study was questionably underpowered to detect a difference in OS, favor using the harder endpoint OS over its surrogate DFS in this study.

   EVIDENCE LEVEL: RCT (HIGH)

7. STAGE IVb DISEASE: Upfront curative intent local treatment without peri-procedural systemic therapy is the appropriate therapy if the surgical procedure, with or without ablative treatment, is considered *complex* (major hepatectomy +/- ablations), with the following two

exemptions where 4-6 cycles of induction systemic therapy are indicated: (a) if downsizing systemic therapy is likely to reduce the surgical risk or (b) in case of early metachronous disease developed within 6 months following primary tumor diagnosis (test of tumor biology) [20-23].

Tanaka et al. reported fewer extended hepatectomies after neoadjuvant chemotherapy. Expert and consensus articles advice peri-procedural chemotherapy for early metachronous disease.

EVIDENCE LEVEL: SEVERAL RETROSPECTIVE COMPARATIVE CASE SERIES AND LARGER REGISTRY RESULTS (LOW)

8. STAGE IVc DISEASE: In patients, unsuitable for curative intent surgery and/or ablation due to number, size and location of CRLM, with potentially downstagable disease, induction systemic therapy is appropriate until: a) curative intent local treatment has become possible or (b) when further downsizing will not (further) decrease procedural risk [24-29].

   Several studies (systematic review, RCTs and retrospective cohort) found significantly increased resectability after induction chemotherapy.

   EVIDENCE LEVEL: RCTs and SYSTEMATIC REVIEWS (HIGH)

9. STAGE IVd DISEASE: For liver-only colorectal metastases, the term permanently unsuitable for curative intent local treatment should be reserved for (a) patients who remain ineligible for radical intent local therapy following induction systemic therapy, (b) patients with upfront contra-indications for radical intent local therapy and contra-indications to receive systemic therapy and (c) patients with a poor general health status who do not qualify for any local therapy (SBRT, local ablation and surgery)[30].

   In the ongoing CAIRO-5 trial, that compares different systemic therapy regimens for unresecatble liver only CRLM patients, a considerable percentage of patients with CRLM originally deemed 'permanently unresectable' were converted to resectable and/or ablatable disease after induction chemotherapy.
   EVIDENCE LEVEL: OBSERVATION FROM RCT, (MODERATE)

10. PROGNOSTIC BIOMARKERS: At the prerequisite that the primary tumor plus any locoregional lymph nodes are (or will be) radically resected (or treated with radical intent otherwise), curative intent local treatment can currently not be (dis)qualified or classified to specific local treatment groups based on the following parameters: primary tumor location, synchronous versus metachronous disease, previous (neo)adjuvant therapies for locoregional disease, the best objectified respons to systemic treatment, (y)p/cT-stage and (y)p/cN-stage, RAS or BRAF wildtypes or mutations, microsatellite (in)stability, consensus molecular subtypes, clinical risk score (CRS by Fong et al.) and the modified CRS, CEA or other tumor marker quantities, the presence and quantity of circulating tumor cells and DNA[31-36].

    The majority of the abovementioned parameters, biomarkers or validated scoring systems have proven to be important prognostic biomarkers correlated with survival, and for some (such as RAS mutation) several studies recommend wider resection and/or ablation margins to prevent recurrence / local tumor progression. However, currently these parameters cannot be used as predictive markers, because they cannot preclude patients from local treatment nor categorize them into a specific local treatment group.

EVIDENCE LEVEL: NO EVIDENCE

11. FUTURE LIVER REMNANT (FLR) VOLUME AND/OR FUNCTION: If the FLR volume and/or function is sufficient, curative-intent local therapy for CRLM is appropriate, regardless of the total number of CRLM; patients cannot be disqualified based on the total number of CRLM alone[37,38].

    Multiple studies have shown long-term disease-free and overall survival after local treatment of CRLM in patients with a high number of CRLM and all suggest superiority of local therapy over chemotherapy alone.

    EVIDENCE LEVEL: SEVERAL RETROSPECTIVE COMPARATIVE CASE SERIES (LOW)

TUMOR CHARACTERISTICS

12. Partial hepatectomy is the appropriate local treatment method for patients with resectable CRLM >3cm, at the prerequisite that ECOG is ≤2, ASA is ≤3 and CCI is ≤8 [39,40].

    Partial hepatectomy is the historical gold standard. Multiple prospective and retrospective series and large scale prospective registries, systematic reviews and meta-analyses have shown a superior overall survival of hepatectomy over systemic therapy alone. Furthermore, long-term disease-free and overall survival and even cure can be established in a substantial proportion of resectable liver only CRLM patients.

    EVIDENCE LEVEL 'PARTIAL HEPATECTOMY SUPERIOR TO SYSTEMIC THERAPY ALONE': (HISTORICAL STANDARD / LOW - MODERATE)

13. Partial hepatectomy is the appropriate local treatment method for patients with resectable *exophytic* or *perihilar* CRLM ≤3cm, at the prerequisite that ECOG is ≤2, ASA is ≤3 and CCI is ≤8 [39,40].

    Partial hepatectomy is the historical gold standard. Multiple prospective and retrospective series and large scale prospective registries, systematic reviews and meta-analyses have shown a superior overall survival of hepatectomy over systemic therapy alone. Furthermore, long-term disease-free and overall survival and even cure can be established in a substantial proportion of resectable liver only CRLM patients.

    EVIDENCE LEVEL 'PARTIAL HEPATECTOMY SUPERIOR TO SYSTEMIC THERAPY ALONE': (HISTORICAL STANDARD / LOW - MODERATE)

14. Partial hepatectomy is the appropriate local treatment method for patients with resectable *superficial or shallow* CRLM ≤3cm, at the prerequisite that the general health condition is <u>good</u> (ECOG ≤1, ASA ≤3 and CCI ≤4) [39-44].

    Partial hepatectomy is the historical gold standard. Multiple prospective and retrospective series and large scale prospective registries, systematic reviews and meta-analyses have shown a superior overall

survival of hepatectomy over systemic therapy alone. Furthermore, long-term disease-free and overall survival and even cure can be established in a substantial proportion of resectable liver only CRLM patients.

EVIDENCE LEVEL 'PARTIAL HEPATECTOMY SUPERIOR TO SYSTEMIC THERAPY ALONE': (HISTORICAL STANDARD / LOW - MODERATE)

Multiple retrospective series, systematic reviews and meta-analyses have shown a superior overall survival of partial hepatectomy over thermal ablation for liver only CRLM. However, given the high risk of residual bias when comparing ablation for unresecatble disease and partial hepatectomy for resectable disease and given several recent series with comparable outcome, the evidence level from these analyses were downgraded.

EVIDENCE LEVEL 'PARTIAL HEPATECTOMY SUPERIOR TO THERMAL ABLATION': (LOW)

15. Thermal ablation can be considered for patients with resectable *superficial or shallow* CRLM ≤3cm, if the general health condition is <u>poor</u> (ECOG 2 and ASA 3 or CCI 5-8)[8,45].

    Patients with a high ECOG, ASA and/or CCI score have a high risk for postoperative mortality and morbidity as mentioned earlier. Abundant series / registries and several systematic reviews and meta-analyses have shown that thermal ablation alone has a very low mortality (≤1%) and low complication rate, even though often performed in patients with suboptimal general health conditions. Given the current clinical equipoise of partial hepatectomy and thermal ablation for small-size CRLM in patients with an optimal general health condition, thermal ablation seems more appropriate in patients with suboptimal general health condition. In other words, the risk-benefit ratio favors thermal ablation over partial hepatectomy in patients with a suboptimal general health status.

    EVIDENCE LEVEL: EXPERT OPINION (LOW)

16. Thermal ablation is the appropriate local treatment method for patients with resectable and thermally ablatable CRLM ≤3cm, if the location of the CRLM is *deep-seated* (e.g. resection would require major hepatectomy), at the prerequisite that ECOG is ≤2, ASA is ≤3 and CCI is ≤8)[46].

    There is no literature available comparing major hepatectomy to thermal ablation. In the COLLISION trial expert panel the majority of the panelists do routinely disagree to randomize these patients. The argumentation being that these patients should be offered ablation due to its lower complication rate, sparing of parenchyma and the good local control rate of thermal ablation. Given the current clinical equipoise of partial hepatectomy and thermal ablation for easily resectable small-size CRLM, thermal ablation seems more appropriate than major hepatectomy for deep-seated CRLM. In other words, the risk-benefit ratio favors thermal ablation over partial hepatectomy in these patients.

    EVIDENCE LEVEL: EXPERT OPINION (LOW)

17. Thermal ablation is the appropriate local treatment method for patients with unresectable and thermally ablatable CRLM ≤3cm, and can be considered for CRLM 3-5cm when (further)

downsizing systemic therapy is unfeasible, at the prerequisite that CCI ≤8, ASA is ≤3 and ECOG is ≤2[21,37,41-44,47].

The long term results of the phase II EORTC CLOCC trial found a superior DFS and OS with the addition of thermal ablation compared to chemotherapy alone in unresectable CRLM, however due to serious indirectness (substantial nr of patients also had resections) and serious imprecision (trial was stopped early, before reaching sample size) the evidence level was downgraded from very high to moderate.

EVIDENCE LEVEL: DOWNGRADED RCT (MODERATE TO HIGH)

18. Irreversible electroporation (IRE) can be considered for patients with unresectable and not thermally ablatable *perihilar* or *perivascular* CRLM ≤3cm, and 3-5cm if further downsizing systemic therapy is unfeasible, at the prerequisite that CCI is ≤8, ASA is ≤3 and ECOG is ≤2[48-50].

Multiple prospective and retrospective studies and a systematic review have found IRE to be a feasible and effective treatment method for unresectable and not thermally ablatable CRLM. The ESMO guidelines have adopted the ablative therapy for CRLM unsuitable for surgery and thermal ablation in 2016. The final results of the prospective COLDFIRE-2 trial are currently under review pending minor residual revisions at the Journal of Clinical Oncology (JCO) and in that phase-2 effectiveness-threshold the primary endpoint was met: 68.0% of patients were alive without local tumor progeression at 1-year post-IRE.

EVIDENCE LEVEL LOW: PROSPECTIVE AND RETROSPECTIVE COHORT STUDIES

19. Stereotactic ablative radiotherapy (SBRT) can be considered for patients with limited disease burden (≤3 CRLM) if an ablative dose can be delivered without jeopardizing liver function and other organs or structures at risk, at the prequisite that ECOG is ≤3, ASA is ≤4 and CCI is ≤10[51-54].

Based on a limited number of studies, liver SBRT, which does not require anesthesia or conscious sedation, seems to be safe and locally effective in select patients with a poor performance status.

EVIDENCE LEVEL LOW: SMALL NON COMPARATIVE CASE SERIES & EXPERT OPINIONS

20. Hemihepatectomy is the appropriate local treatment method for multiple CRLM (≥3) within a single lobe when at least one of these CRLM is deep-seated, even when potentially ablatable, at the prerequisite that CCI is ≤8, ASA is ≤3 and ECOG is ≤2[39,40].

There is no literature available comparing major hepatectomy to multiple single-lobe thermal ablations. In the COLLISION trial expert panel the majority of the panelists do routinely disagree to randomize these patients. They should be offered (major) hepatectomy. The abovementioned argument to favor thermal ablation over major hepatectomy for deep-seated CRLM given its low complication rate and good local control is less apparent when multiple tumors have to be ablated within a single lobe. In other words, the risk-benefit ratio does not favor multiple unilobar thermal ablations over hemi-hepatectomy in this subgroup.

EVIDENCE LEVEL VERY LOW: SMALL NON COMPARATIVE CASE SERIES & EXPERT OPINIONS

21. When considering fit patients with multiple *scattered* and *bilobar* CRLM ≤3cm (≥6 CRLM in total and ≥3 deep-seated CRLM in both lobes separately) what treatment is appropriate: *chip-and-burn* wedge resection(s) of all exophytic, superficial and shallow CRLM and thermal ablation of all deep-seated CRLM; or a *2-stage-hepatectomy*: stage 1: wedge resection(s) of all superficial CRLM and thermal ablations of all deep-seated CRLM in 1 lobe and (following contralateral liver augmentation) stage 2: contralateral hemihepatectomy[55,56].

    Score 1-3: chip-and-burn
    Score 4-6: equipoise
    Score 7-9: 2-stage-hepatectomy

    A systematic review on two-stage hepatectomy showed median OS of 37 months, this is comparable to the results of a study on single-stage resection + MWA showing a median OS of 38-42 months)

    EVIDENCE LEVEL LOW: NON COMPARATIVE CASE SERIES & EXPERT OPINIONS

TREATMENT CHARACTERISTICS

22. Anatomical contra-indications for partial hepatectomy are: (a) inability to obtain R0 margins, (b) inability to leave a sufficient FLR volume and/or function, (c) inability to preserve the dual blood supply and the venous and biliary drainage from the FLR and (d) inaccessibility of the abdominal cavity due to excessive abdominal adhesions[18,50,57,58].

    Definition adheres to several previously published attempts to postulate resectability criteria.

23. Anatomical contra-indications for thermal ablation are: (a) peri-tumoral vicinity (<10mm) of the common, left or right hepatic bile duct or (b) peri-hepatic critical structures that cannot be distanced using surgical or interventional dissection methods, (c) the abutment or encasement of the single remaining major portal or systemic vein following surgery and (d) an invasion of the free wall of the inferior caval vein. The maximum size is 3cm, although thermal ablation can be considered for 3-5cm unresectable CRLM after failure to (further) downsize them with systemic therapy[59].

    Definition adheres to the quality improvement guidelines published by CIRSE.

24. Contra-indications for irreversible electroporation are: CRLM >5cm, ventricular arrhythmias, cardiac stimulation devices and congestive heart failure.

    Definition adheres to the standardized protocol published in JVIR 2020: Irreversible electroporation for hepatic tumors: protocol standardization using the modified Delphi technique. Ruarus et al. JVIR 2020

25. Contra-indications for stereotactic body radiotherapy (SBRT) are: >3 CRLM and inability do deliver an ablative dose without jeopardizing liver function and adjacent organs or structures at risk[51,60].

References

1. Brudvik, K.W.; Rosok, B.; Naresh, U.; Yaqub, S.; Fretland, A.A.; Labori, K.J.; Edwin, B.; Bjornbeth, B.A. Survival after resection of colorectal liver metastases in octogenarians and sexagenarians compared to their respective age-matched national population. *Hepatobiliary Surg Nutr* **2018**, *7*, 234-241, doi:10.21037/hbsn.2017.09.03.
2. Grande, R.; Natoli, C.; Ciancola, F.; Gemma, D.; Pellegrino, A.; Pavese, I.; Garufi, C.; Di Lauro, L.; Corsi, D.; Signorelli, D., et al. Treatment of Metastatic Colorectal Cancer Patients >/=75 Years Old in Clinical Practice: A Multicenter Analysis. *PLoS One* **2016**, *11*, e0157751, doi:10.1371/journal.pone.0157751.
3. Kulik, U.; Framke, T.; Grosshennig, A.; Ceylan, A.; Bektas, H.; Klempnauer, J.; Lehner, F. Liver resection of colorectal liver metastases in elderly patients. *World J Surg* **2011**, *35*, 2063-2072, doi:10.1007/s00268-011-1180-x.
4. Booth, C.M.; Nanji, S.; Wei, X.; Mackillop, W.J. Management and Outcome of Colorectal Cancer Liver Metastases in Elderly Patients: A Population-Based Study. *JAMA Oncol* **2015**, *1*, 1111-1119, doi:10.1001/jamaoncol.2015.2943.
5. Stillwell, A.P.; Ho, Y.H.; Veitch, C. Systematic review of prognostic factors related to overall survival in patients with stage IV colorectal cancer and unresectable metastases. *World J Surg* **2011**, *35*, 684-692, doi:10.1007/s00268-010-0891-8.
6. Rocha, L.; Riechelmann, R.P. Treatment of patients with metastatic colorectal cancer and poor performance status: current evidence and challenges. *Clinics (Sao Paulo)* **2018**, *73*, e542s, doi:10.6061/clinics/2018/e542s.
7. Sargent, D.J.; Kohne, C.H.; Sanoff, H.K.; Bot, B.M.; Seymour, M.T.; de Gramont, A.; Porschen, R.; Saltz, L.B.; Rougier, P.; Tournigand, C., et al. Pooled safety and efficacy analysis examining the effect of performance status on outcomes in nine first-line treatment trials using individual data from patients with metastatic colorectal cancer. *J Clin Oncol* **2009**, *27*, 1948-1955, doi:10.1200/JCO.2008.20.2879.
8. Chou, W.C.; Liu, K.H.; Lu, C.H.; Hung, Y.S.; Chen, M.F.; Cheng, Y.F.; Wang, C.H.; Lin, Y.C.; Yeh, T.S. To Operate or Not: Prediction of 3-Month Postoperative Mortality in Geriatric Cancer Patients. *J Cancer* **2016**, *7*, 14-21, doi:10.7150/jca.13126.
9. Kok, E.N.D.; Jansen, E.P.M.; Heeres, B.C.; Kok, N.F.M.; Janssen, T.; van Werkhoven, E.; Sanders, F.R.K.; Ruers, T.J.M.; Nowee, M.E.; Kuhlmann, K.F.D. High versus low dose Stereotactic Body Radiation Therapy for hepatic metastases. *Clin Transl Radiat Oncol* **2020**, *20*, 45-50, doi:10.1016/j.ctro.2019.11.004.
10. Zemin He, G.C., Bo Ouyang. Conformal Radiation Therapy or Stereotactic Body Radiation Therapy: Institutional Experience in the Management of Colorectal Liver Metastases by Radiation Therapy. *Technology in Cancer Research & Treatment* **2018**, *17*.
11. Guckenberger, M.; Andratschke, N.; Dieckmann, K.; Hoogeman, M.S.; Hoyer, M.; Hurkmans, C.; Tanadini-Lang, S.; Lartigau, E.; Mendez Romero, A.; Senan, S., et al. ESTRO ACROP consensus guideline on implementation and practice of stereotactic body radiotherapy for peripherally located early stage non-small cell lung cancer. *Radiother Oncol* **2017**, *124*, 11-17, doi:10.1016/j.radonc.2017.05.012.
12. Schroeder, R.A.; Marroquin, C.E.; Bute, B.P.; Khuri, S.; Henderson, W.G.; Kuo, P.C. Predictive indices of morbidity and mortality after liver resection. *Ann Surg* **2006**, *243*, 373-379, doi:10.1097/01.sla.0000201483.95911.08.
13. Tzeng, C.W.; Cooper, A.B.; Vauthey, J.N.; Curley, S.A.; Aloia, T.A. Predictors of morbidity and mortality after hepatectomy in elderly patients: analysis of 7621 NSQIP patients. *HPB (Oxford)* **2014**, *16*, 459-468, doi:10.1111/hpb.12155.
14. Friedman, L.S. Surgery in the patient with liver disease. *Trans Am Clin Climatol Assoc* **2010**, *121*, 192-204; discussion 205.

15. Forner, A.; Llovet, J.M.; Bruix, J. Hepatocellular carcinoma. *Lancet* **2012**, *379*, 1245-1255, doi:10.1016/S0140-6736(11)61347-0.
16. Gooiker, G.A.; Dekker, J.W.; Bastiaannet, E.; van der Geest, L.G.; Merkus, J.W.; van de Velde, C.J.; Tollenaar, R.A.; Liefers, G.J. Risk factors for excess mortality in the first year after curative surgery for colorectal cancer. *Ann Surg Oncol* **2012**, *19*, 2428-2434, doi:10.1245/s10434-012-2294-6.
17. Simons, J.P.; Ng, S.C.; Hill, J.S.; Shah, S.A.; Bodnari, A.; Zhou, Z.; Tseng, J.F. In-hospital mortality for liver resection for metastases: a simple risk score. *J Surg Res* **2009**, *156*, 21-25, doi:10.1016/j.jss.2009.03.073.
18. Nordlinger, B.; Sorbye, H.; Glimelius, B.; Poston, G.J.; Schlag, P.M.; Rougier, P.; Bechstein, W.O.; Primrose, J.N.; Walpole, E.T.; Finch-Jones, M., et al. Perioperative chemotherapy with FOLFOX4 and surgery versus surgery alone for resectable liver metastases from colorectal cancer (EORTC Intergroup trial 40983): a randomised controlled trial. *Lancet* **2008**, *371*, 1007-1016, doi:10.1016/S0140-6736(08)60455-9.
19. Nordlinger, B.; Sorbye, H.; Glimelius, B.; Poston, G.J.; Schlag, P.M.; Rougier, P.; Bechstein, W.O.; Primrose, J.N.; Walpole, E.T.; Finch-Jones, M., et al. Perioperative FOLFOX4 chemotherapy and surgery versus surgery alone for resectable liver metastases from colorectal cancer (EORTC 40983): long-term results of a randomised, controlled, phase 3 trial. *Lancet Oncol* **2013**, *14*, 1208-1215, doi:10.1016/S1470-2045(13)70447-9.
20. Tanaka, K.; Adam, R.; Shimada, H.; Azoulay, D.; Levi, F.; Bismuth, H. Role of neoadjuvant chemotherapy in the treatment of multiple colorectal metastases to the liver. *Br J Surg* **2003**, *90*, 963-969, doi:10.1002/bjs.4160.
21. Adam, R.; de Gramont, A.; Figueras, J.; Kokudo, N.; Kunstlinger, F.; Loyer, E.; Poston, G.; Rougier, P.; Rubbia-Brandt, L.; Sobrero, A., et al. Managing synchronous liver metastases from colorectal cancer: a multidisciplinary international consensus. *Cancer Treat Rev* **2015**, *41*, 729-741, doi:10.1016/j.ctrv.2015.06.006.
22. Allen, P.J.; Kemeny, N.; Jarnagin, W.; DeMatteo, R.; Blumgart, L.; Fong, Y. Importance of response to neoadjuvant chemotherapy in patients undergoing resection of synchronous colorectal liver metastases. *J Gastrointest Surg* **2003**, *7*, 109-117, doi:10.1016/S1091-255X(02)00121-X.
23. Reddy, S.K.; Zorzi, D.; Lum, Y.W.; Barbas, A.S.; Pawlik, T.M.; Ribero, D.; Abdalla, E.K.; Choti, M.A.; Kemp, C.; Vauthey, J.N., et al. Timing of multimodality therapy for resectable synchronous colorectal liver metastases: a retrospective multi-institutional analysis. *Ann Surg Oncol* **2009**, *16*, 1809-1819, doi:10.1245/s10434-008-0181-y.
24. Folprecht, G.; Gruenberger, T.; Bechstein, W.; Raab, H.R.; Weitz, J.; Lordick, F.; Hartmann, J.T.; Stoehlmacher-Williams, J.; Lang, H.; Trarbach, T., et al. Survival of patients with initially unresectable colorectal liver metastases treated with FOLFOX/cetuximab or FOLFIRI/cetuximab in a multidisciplinary concept (CELIM study). *Ann Oncol* **2014**, *25*, 1018-1025, doi:10.1093/annonc/mdu088.
25. Adam, R.; Pascal, G.; Castaing, D.; Azoulay, D.; Delvart, V.; Paule, B.; Levi, F.; Bismuth, H. Tumor progression while on chemotherapy: a contraindication to liver resection for multiple colorectal metastases? *Ann Surg* **2004**, *240*, 1052-1061; discussion 1061-1054, doi:10.1097/01.sla.0000145964.08365.01.
26. Malik, H.; Khan, A.Z.; Berry, D.P.; Cameron, I.C.; Pope, I.; Sherlock, D.; Helmy, S.; Byrne, B.; Thompson, M.; Pulfer, A., et al. Liver resection rate following downsizing chemotherapy with cetuximab in metastatic colorectal cancer: UK retrospective observational study. *Eur J Surg Oncol* **2015**, *41*, 499-505, doi:10.1016/j.ejso.2015.01.032.
27. Tomasello, G.; Petrelli, F.; Ghidini, M.; Russo, A.; Passalacqua, R.; Barni, S. FOLFOXIRI Plus Bevacizumab as Conversion Therapy for Patients With Initially Unresectable Metastatic Colorectal Cancer: A Systematic Review and Pooled Analysis. *JAMA Oncol* **2017**, *3*, e170278, doi:10.1001/jamaoncol.2017.0278.

28. Folprecht, G.; Gruenberger, T.; Bechstein, W.O.; Raab, H.R.; Lordick, F.; Hartmann, J.T.; Lang, H.; Frilling, A.; Stoehlmacher, J.; Weitz, J., et al. Tumour response and secondary resectability of colorectal liver metastases following neoadjuvant chemotherapy with cetuximab: the CELIM randomised phase 2 trial. *Lancet Oncol* **2010**, *11*, 38-47, doi:10.1016/S1470-2045(09)70330-4.
29. Ye, L.C.; Liu, T.S.; Ren, L.; Wei, Y.; Zhu, D.X.; Zai, S.Y.; Ye, Q.H.; Yu, Y.; Xu, B.; Qin, X.Y., et al. Randomized controlled trial of cetuximab plus chemotherapy for patients with KRAS wild-type unresectable colorectal liver-limited metastases. *J Clin Oncol* **2013**, *31*, 1931-1938, doi:10.1200/JCO.2012.44.8308.
30. Huiskens, J.; Bolhuis, K.; Engelbrecht, M.R.; De Jong, K.P.; Kazemier, G.; Liem, M.S.; Verhoef, C.; de Wilt, J.H.; Punt, C.J.; van Gulik, T.M., et al. Outcomes of Resectability Assessment of the Dutch Colorectal Cancer Group Liver Metastases Expert Panel. *J Am Coll Surg* **2019**, *229*, 523-532 e522, doi:10.1016/j.jamcollsurg.2019.08.1445.
31. Guinney, J.; Dienstmann, R.; Wang, X.; de Reynies, A.; Schlicker, A.; Soneson, C.; Marisa, L.; Roepman, P.; Nyamundanda, G.; Angelino, P., et al. The consensus molecular subtypes of colorectal cancer. *Nat Med* **2015**, *21*, 1350-1356, doi:10.1038/nm.3967.
32. Brudvik, K.W.; Jones, R.P.; Giuliante, F.; Shindoh, J.; Passot, G.; Chung, M.H.; Song, J.; Li, L.; Dagenborg, V.J.; Fretland, A.A., et al. RAS Mutation Clinical Risk Score to Predict Survival After Resection of Colorectal Liver Metastases. *Ann Surg* **2019**, *269*, 120-126, doi:10.1097/SLA.0000000000002319.
33. Brudvik, K.W.; Shindoh, J. Limitations of molecular biomarkers in patients with resectable colorectal liver metastases. *Chin Clin Oncol* **2019**, *8*, 48, doi:10.21037/cco.2019.08.02.
34. Fong, Y.; Fortner, J.; Sun, R.L.; Brennan, M.F.; Blumgart, L.H. Clinical score for predicting recurrence after hepatic resection for metastatic colorectal cancer: analysis of 1001 consecutive cases. *Ann Surg* **1999**, *230*, 309-318; discussion 318-321, doi:10.1097/00000658-199909000-00004.
35. Tsilimigras, D.I.; Ntanasis-Stathopoulos, I.; Bagante, F.; Moris, D.; Cloyd, J.; Spartalis, E.; Pawlik, T.M. Clinical significance and prognostic relevance of KRAS, BRAF, PI3K and TP53 genetic mutation analysis for resectable and unresectable colorectal liver metastases: A systematic review of the current evidence. *Surg Oncol* **2018**, *27*, 280-288, doi:10.1016/j.suronc.2018.05.012.
36. Turano, M.; Delrio, P.; Rega, D.; Cammarota, F.; Polverino, A.; Duraturo, F.; Izzo, P.; De Rosa, M. Promising Colorectal Cancer Biomarkers for Precision Prevention and Therapy. *Cancers (Basel)* **2019**, *11*, doi:10.3390/cancers11121932.
37. Ruers, T.; Van Coevorden, F.; Punt, C.J.; Pierie, J.E.; Borel-Rinkes, I.; Ledermann, J.A.; Poston, G.; Bechstein, W.; Lentz, M.A.; Mauer, M., et al. Local Treatment of Unresectable Colorectal Liver Metastases: Results of a Randomized Phase II Trial. *J Natl Cancer Inst* **2017**, *109*, doi:10.1093/jnci/djx015.
38. Allard, M.A.; Adam, R.; Giuliante, F.; Lapointe, R.; Hubert, C.; Ijzermans, J.N.M.; Mirza, D.F.; Elias, D.; Laurent, C.; Gruenberger, T., et al. Long-term outcomes of patients with 10 or more colorectal liver metastases. *Br J Cancer* **2017**, *117*, 604-611, doi:10.1038/bjc.2017.218.
39. Creasy, J.M.; Sadot, E.; Koerkamp, B.G.; Chou, J.F.; Gonen, M.; Kemeny, N.E.; Balachandran, V.P.; Kingham, T.P.; DeMatteo, R.P.; Allen, P.J., et al. Actual 10-year survival after hepatic resection of colorectal liver metastases: what factors preclude cure? *Surgery* **2018**, *163*, 1238-1244, doi:10.1016/j.surg.2018.01.004.
40. Kanas, G.P.; Taylor, A.; Primrose, J.N.; Langeberg, W.J.; Kelsh, M.A.; Mowat, F.S.; Alexander, D.D.; Choti, M.A.; Poston, G. Survival after liver resection in metastatic colorectal cancer: review and meta-analysis of prognostic factors. *Clin Epidemiol* **2012**, *4*, 283-301, doi:10.2147/CLEP.S34285.
41. Tinguely, P.; Dal, G.; Bottai, M.; Nilsson, H.; Freedman, J.; Engstrand, J. Microwave ablation versus resection for colorectal cancer liver metastases - A propensity score analysis from a

population-based nationwide registry. *Eur J Surg Oncol* **2020**, *46*, 476-485, doi:10.1016/j.ejso.2019.12.002.
42. Hof, J.; Wertenbroek, M.W.; Peeters, P.M.; Widder, J.; Sieders, E.; de Jong, K.P. Outcomes after resection and/or radiofrequency ablation for recurrence after treatment of colorectal liver metastases. *Br J Surg* **2016**, *103*, 1055-1062, doi:10.1002/bjs.10162.
43. van Amerongen, M.J.; Jenniskens, S.F.M.; van den Boezem, P.B.; Futterer, J.J.; de Wilt, J.H.W. Radiofrequency ablation compared to surgical resection for curative treatment of patients with colorectal liver metastases - a meta-analysis. *HPB (Oxford)* **2017**, *19*, 749-756, doi:10.1016/j.hpb.2017.05.011.
44. Meijerink, M.R.; Puijk, R.S.; van Tilborg, A.; Henningsen, K.H.; Fernandez, L.G.; Neyt, M.; Heymans, J.; Frankema, J.S.; de Jong, K.P.; Richel, D.J., et al. Radiofrequency and Microwave Ablation Compared to Systemic Chemotherapy and to Partial Hepatectomy in the Treatment of Colorectal Liver Metastases: A Systematic Review and Meta-Analysis. *Cardiovasc Intervent Radiol* **2018**, *41*, 1189-1204, doi:10.1007/s00270-018-1959-3.
45. Lahat, E.; Eshkenazy, R.; Zendel, A.; Zakai, B.B.; Maor, M.; Dreznik, Y.; Ariche, A. Complications after percutaneous ablation of liver tumors: a systematic review. *Hepatobiliary Surg Nutr* **2014**, *3*, 317-323, doi:10.3978/j.issn.2304-3881.2014.09.07.
46. Takahashi, H.; Berber, E. Role of thermal ablation in the management of colorectal liver metastasis. *Hepatobiliary Surg Nutr* **2020**, *9*, 49-58, doi:10.21037/hbsn.2019.06.08.
47. Ruers, T.; Punt, C.; Van Coevorden, F.; Pierie, J.P.; Borel-Rinkes, I.; Ledermann, J.A.; Poston, G.; Bechstein, W.; Lentz, M.A.; Mauer, M., et al. Radiofrequency ablation combined with systemic treatment versus systemic treatment alone in patients with non-resectable colorectal liver metastases: a randomized EORTC Intergroup phase II study (EORTC 40004). *Ann Oncol* **2012**, *23*, 2619-2626, doi:10.1093/annonc/mds053.
48. Schicho, A.; Niessen, C.; Haimerl, M.; Wiesinger, I.; Stroszczynski, C.; Beyer, L.P.; Wiggermann, P. Long-term survival after percutaneous irreversible electroporation of inoperable colorectal liver metastases. *Cancer Manag Res* **2019**, *11*, 317-322, doi:10.2147/CMAR.S182091.
49. Ruarus, A.H.; Vroomen, L.; Puijk, R.S.; Scheffer, H.J.; Zonderhuis, B.M.; Kazemier, G.; van den Tol, M.P.; Berger, F.H.; Meijerink, M.R. Irreversible Electroporation in Hepatopancreaticobiliary Tumours. *Can Assoc Radiol J* **2018**, *69*, 38-50, doi:10.1016/j.carj.2017.10.005.
50. Van Cutsem, E.; Cervantes, A.; Adam, R.; Sobrero, A.; Van Krieken, J.H.; Aderka, D.; Aranda Aguilar, E.; Bardelli, A.; Benson, A.; Bodoky, G., et al. ESMO consensus guidelines for the management of patients with metastatic colorectal cancer. *Ann Oncol* **2016**, *27*, 1386-1422, doi:10.1093/annonc/mdw235.
51. Petrelli, F.; Comito, T.; Barni, S.; Pancera, G.; Scorsetti, M.; Ghidini, A.; metastases, S.f.C.l. Stereotactic body radiotherapy for colorectal cancer liver metastases: A systematic review. *Radiother Oncol* **2018**, *129*, 427-434, doi:10.1016/j.radonc.2018.06.035.
52. Mahadevan, A.; Blanck, O.; Lanciano, R.; Peddada, A.; Sundararaman, S.; D'Ambrosio, D.; Sharma, S.; Perry, D.; Kolker, J.; Davis, J. Stereotactic Body Radiotherapy (SBRT) for liver metastasis - clinical outcomes from the international multi-institutional RSSearch(R) Patient Registry. *Radiat Oncol* **2018**, *13*, 26, doi:10.1186/s13014-018-0969-2.
53. Andratschke, N.; Alheid, H.; Allgauer, M.; Becker, G.; Blanck, O.; Boda-Heggemann, J.; Brunner, T.; Duma, M.; Gerum, S.; Guckenberger, M., et al. The SBRT database initiative of the German Society for Radiation Oncology (DEGRO): patterns of care and outcome analysis of stereotactic body radiotherapy (SBRT) for liver oligometastases in 474 patients with 623 metastases. *BMC Cancer* **2018**, *18*, 283, doi:10.1186/s12885-018-4191-2.
54. Scorsetti, M.; Comito, T.; Clerici, E.; Franzese, C.; Tozzi, A.; Iftode, C.; Di Brina, L.; Navarria, P.; Mancosu, P.; Reggiori, G., et al. Phase II trial on SBRT for unresectable liver metastases:

long-term outcome and prognostic factors of survival after 5 years of follow-up. *Radiat Oncol* **2018**, *13*, 234, doi:10.1186/s13014-018-1185-9.
55. Philips, P.; Groeschl, R.T.; Hanna, E.M.; Swan, R.Z.; Turaga, K.K.; Martinie, J.B.; Iannitti, D.A.; Schmidt, C.; Gamblin, T.C.; Martin, R.C. Single-stage resection and microwave ablation for bilobar colorectal liver metastases. *Br J Surg* **2016**, *103*, 1048-1054, doi:10.1002/bjs.10159.
56. Lam, V.W.; Laurence, J.M.; Johnston, E.; Hollands, M.J.; Pleass, H.C.; Richardson, A.J. A systematic review of two-stage hepatectomy in patients with initially unresectable colorectal liver metastases. *HPB (Oxford)* **2013**, *15*, 483-491, doi:10.1111/j.1477-2574.2012.00607.x.
57. Nordlinger, B.; Van Cutsem, E.; Gruenberger, T.; Glimelius, B.; Poston, G.; Rougier, P.; Sobrero, A.; Ychou, M.; European Colorectal Metastases Treatment, G.; Sixth International Colorectal Liver Metastases, W. Combination of surgery and chemotherapy and the role of targeted agents in the treatment of patients with colorectal liver metastases: recommendations from an expert panel. *Ann Oncol* **2009**, *20*, 985-992, doi:10.1093/annonc/mdn735.
58. Adams, R.B.; Aloia, T.A.; Loyer, E.; Pawlik, T.M.; Taouli, B.; Vauthey, J.N.; Americas Hepato-Pancreato-Biliary, A.; Society of Surgical, O.; Society for Surgery of the Alimentary, T. Selection for hepatic resection of colorectal liver metastases: expert consensus statement. *HPB (Oxford)* **2013**, *15*, 91-103, doi:10.1111/j.1477-2574.2012.00557.x.
59. Crocetti, L.; de Baere, T.; Lencioni, R. Quality improvement guidelines for radiofrequency ablation of liver tumours. *Cardiovasc Intervent Radiol* **2010**, *33*, 11-17, doi:10.1007/s00270-009-9736-y.
60. Mendez Romero, A.; Keskin-Cambay, F.; van Os, R.M.; Nuyttens, J.J.; Heijmen, B.J.M.; JNM, I.J.; Verhoef, C. Institutional experience in the treatment of colorectal liver metastases with stereotactic body radiation therapy. *Rep Pract Oncol Radiother* **2017**, *22*, 126-131, doi:10.1016/j.rpor.2016.10.003.
