# Supplementary material for: Resectability and Ablatability Criteria for the Treatment of Liver Only Colorectal Metastases: Multidisciplinary Consensus Document from the COLLISION Trial Group
Source: Cancers (Basel). 2020 Jul 3;12(7):1779. doi: 10.3390/cancers12071779 (PMC7407587; doi:10.3390/cancers12071779)
Supplement: Supplementary file 1 [file cancers-12-01779-s001.zip › File S2 - definitions.pdf]

Online appendix B: Definitions

Eastern Cooperative Oncology Group (ECOG) performance status

0	Fully active, able to carry on all pre-disease performance without restriction
1	Restricted in physically strenuous activity but ambulatory and able to carry out work of a light or sedentary nature, e.g., light house work, office work
2	Ambulatory and capable of all selfcare but unable to carry out any work activities; up and about more than 50% of waking hours
3	Capable of only limited selfcare; confined to bed or chair more than 50% of waking hours
4	Completely disabled; cannot carry on any selfcare; totally confined to bed or chair
5	Dead

American Society of Anesthesiologists (ASA) performance status

ASA I	A normal healthy patient
ASA II	A patient with mild systemic disease. Mild diseases only without substantive functional limitations.
ASA III	A patient with severe systemic disease. Substantive functional limitations; One or more moderate to severe diseases.
ASA IV	A patient with severe systemic disease that is a constant threat to life.
ASA V	A moribund patient who is not expected to survive without the operation
ASA VI	A declared brain-dead patient whose organs are being removed for donor purposes

Underlying liver disease

Mild liver disease = chronic hepatitis (or cirrhosis without portal hypertension)

Severe liver disease = cirrhosis and portal hypertension with or without variceal bleeding history

Charlson Comorbidity Index (CCI)

Variable	Definition	Points
Myocardial infarction	History of definite or probable MI (EKG changes and/or enzyme changes)	1
Congestive heart failure	Exertional or paroxysmal nocturnal dyspnea and has responded to digitalis, diuretics, or afterload reducing agents	1
Peripheral vascular disease	Intermittent claudication or past bypass for chronic arterial insufficiency, history of gangrene or acute arterial insufficiency, or untreated thoracic or abdominal aneurysm (≥6 cm)	1
Cerebrovascular accident or transient ischemic attack	History of a cerebrovascular accident with minor or no residua and transient ischemic attacks	1
Dementia	Chronic cognitive deficit	1
Chronic obstructive pulmonary disease	-	1
Connective tissue disease	-	1
Peptic ulcer disease	Any history of treatment for ulcer disease or history of ulcer bleeding	1
Mild liver disease*	Mild = chronic hepatitis (or cirrhosis without portal hypertension)	1
Uncomplicated diabetes*	-	1
Hemiplegia	-	2
Moderate to severe chronic kidney disease	Severe = on dialysis, status post kidney transplant, uremia, moderate = creatinine >3 mg/dL (0.27 mmol/L)	2
Diabetes with end-organ damage*	-	2
Localized solid tumor**	-	2
Leukemia	-	2
Lymphoma	-	2
Moderate to severe liver disease*	Severe = cirrhosis and portal hypertension with variceal bleeding history, moderate = cirrhosis and portal hypertension but no variceal bleeding history	3

Metastatic cancer**	-	6
AIDS	-	6

Plus 1 point for every decade age 50 years and over, maximum 4 points

* liver disease and diabetes inputs are mutually exclusive (e.g. do not give points for both "mild liver disease" and "moderate or severe liver disease").

** Other than the colorectal cancer for which the patient is currently being assessed (the comorbidity score is for co-existing diseases)

General health status

Very poor general health status: ECOG ≥3, ASA ≥4 and/or CCI ≥9

Poor general health status: ECOG 2 *and* ASA 3 or CCI 5-8

Good general health status: ECOG ≤2, ASA ≤3 and CCI ≤4

Fong Clinical Risk Score (CRS)

Validated prognostic score for CRLM

Node-positive primary	1 point
Disease -free interval from primary to metastases <12 months	1 point
Number of hepatic tumors >1	1 point
Largest hepatic tumor >5cm	1 point
carcinoembryonic antigen level >200 ng/ml	1 point

0-2 points low risk

3-5 points high risk

Note: CRS is defined only once, at the time of detection of the CRLM

Hepatectomy

No consensus exists regarding the definition of a major hepatectomy and more specifically the minimum number of segments removed. Because this is off topic for the current study, we avoided

the ‘number of segments removed’ discussion and classified resection types into minor versus major, hereby adhering to both the Dutch DHBA guidelines and the COLLISION trial research protocol.

A standard definition of major hepatectomy: resection of four or more liver segments. Srinevas K. Reddy et al. HPB 2011; Redefining major hepatic resection for colorectal liver metastases: Analysis of 1111 liver resections. Gareth Morris-Stiff et al. Int J Surg 2016; Implementation and first results of a mandatory, nationwide audit on liver surgery; Implementation and first results of a mandatory, nationwide audit on liver surgery. Leonie R. van der Werf et al. HPB 2016.

Minor hepatectomy: wedge resection, segmentectomy, left lateral sectionectomy, right posterior bisectionectomy, residual healthy liver volume >40%, ≤1 hepatic vein involved <u>and</u> inferior caval vein free from tumor

Major hepatectomy: (extended) hemihepatectomy, left medial sectionectomy (sIV), right anterior sectionectomy (sV/VIII), central bisegmentectomy (sIV/V /VIII), residual healthy liver volume ≤40%, <u>or</u> biliary or vascular reconstruction(s) required

<u>Future liver remnant</u>

Future liver remnant volume is defined as the ratio of the remnant functioning liver volume (FLR) to the total functional liver volume (TFLV). The TLV is calculated using the following formula: total liver volume (TLV) – tumor volume (TV) = TFLV. Future liver remnant function is calculated using ^{99}mTc-mebrofenin hepatobiliary scintigraphy. There is currently no consensus whether to prefer FLR volume, FLR function or both and hence this is up to local expertise and will be disregarded in our paper as it is off-topic.

<u>Early metachronous disease</u>

Occurrence of CRLM within 6 months after diagnosis of the primary cancer in patients without metastases at the time of diagnosis of the primary tumor, at the prerequisite that adequate cross-sectional imaging for staging purposes was performed at baseline.

<u>Perihilar CRLM</u>

‘Involvement (direct abutment, ingrowth or encasement) of the central bile ducts’ here means resection would require biliary reconstruction surgery if resected.

Peritumoral vicinity (<10 mm) of the central bile ducts here means thermal ablation is contra-indicated.

<u>Perivascular CRLM</u>

‘Involvement (direct abutment, ingrowth or encasement) of major blood vessels’ here means resection would require vascular reconstruction and thermal ablation would entail risk of vascular thrombosis, occlusion and / or life-threatening hemorrhage.

‘Peritumoral vicinity of major blood vessels’ here means thermal ablation is not contra-indicated, but tumors are at risk for heat-sink induced incomplete ablations.

The following location definitions *only apply to* small-size CRLM (≤3cm)!

<u>Exophytic CRLM</u>

The center or at least a substantial part of the tumor lies beyond the confines of the liver.

<u>Superficial CRLM</u>

Tumors located at the surface of the liver that require minor hepatectomy.

<u>Shallow CRLM</u>

Tumors located sub-surface or at shallow depths that require minor hepatectomy.

<u>Deep-seated CRLM</u>

Deep-seated CRLM that, by definition, require major hepatectomy.
